# Altered neural activity to monetary reward/loss processing in episodic migraine

**DOI:** 10.1038/s41598-019-41867-x

**Published:** 2019-04-01

**Authors:** Natália Kocsel, Attila Galambos, Edina Szabó, Andrea Edit Édes, Máté Magyar, Terézia Zsombók, Dorottya Pap, Lajos Rudolf Kozák, György Bagdy, Gyöngyi Kökönyei, Gabriella Juhász

**Affiliations:** 1 Doctoral School of Psychology, ELTE Eötvös Loránd University, Budapest, Hungary; 2 Institute of Psychology, ELTE Eötvös Loránd University, Budapest, Hungary; 3 SE-NAP2 Genetic Brain Imaging Migraine Research Group, Semmelweis University, Budapest, Hungary; 4 Department of Pharmacodynamics, Faculty of Pharmacy, Semmelweis University, Budapest, Hungary; 5 MTA-SE Neuropsychopharmacology and Neurochemistry Research Group, Hungarian Academy of Sciences, Semmelweis University, Budapest, Hungary; 6 Department of Neurology, Faculty of Medicine, Semmelweis University, Budapest, Hungary; 7 MR Research Center, Semmelweis University, Budapest, Hungary; 8 Neuroscience and Psychiatry Unit, The University of Manchester and Manchester Academic Health Sciences Centre, Manchester, United Kingdom

## Abstract

The dysfunctions of the mesolimbic cortical reward circuit have been proposed to contribute to migraine pain. Although supporting empirical evidence was mainly found in connection with primary rewards or in chronic migraine where the pain experience is (almost) constant. Our goal however was to investigate the neural correlates of secondary reward/loss anticipation and consumption using the monetary incentive delay task in 29 episodic migraine patients and 41 headache-free controls. Migraine patients showed decreased activation in one cluster covering the right inferior frontal gyrus during reward consumption compared to controls. We also found significant negative correlation between the time of the last migraine attack before the scan and activation of the parahippocampal gyrus and the right hippocampus yielded to loss anticipation. During reward/loss consumption, a relative increase in the activity of the visual areas was observed the more time passed between the last attack and the scan session. Our results suggest intact reward/loss anticipation but altered reward consumption in migraine, indicating a decreased reactivity to monetary rewards. The findings also raise the possibility that neural responses to loss anticipation and reward/loss consumption could be altered by the proximity of the last migraine attack not just during pre-ictal periods, but interictally as well.

## Introduction

Migraine is a massively common and impairing primary headache disorder, which was ranked the third highest cause of disability among the population under 50 years of age by the Global Burden of Disease studies (GBD, 2015)^1^.

It is conceptualized as a multiphasic neurobiological disorder, which is characterized by various accompanying symptoms besides pain, such as phonophobia, photophobia, nausea, vomiting or cutaneous allodynia^2–4^. Although these symptoms often occur simultaneously with headache^5^, several behavioural, affective and cognitive symptoms exist^3,6^ which follow a specific sequence over time and start days before^7^ or after the migraine attack^2,7^. Changes in appetite or in mood, yawning or fatigue typically precede the attacks, while, for example fatigue, tiredness, euphoria or dysphoria frequently follow the headache. Based on these observations, Blau^5^ and May^3^ presumed, that migraine is not an isolated event but a continuous oscillation of various bodily and sensory functions, where the pain itself is just one of the symptoms.

Several findings of advanced neuroimaging supported this assumption^8,9^, identifying brain structural and functional alterations in migraineurs compared to healthy controls, not just during migraine attacks^4^, but in interictal stages too^10^. Interictal imaging showed for example reductions in frontal and parietal lobe density along with an executive function deficit in set-shifting task^11^; and an enhanced reactivity of the visual cortex after visual stimulation^12^. These alterations are often positively correlated with disease duration^13^ and headache frequency^14^. In addition, Schwedt *et al*.^15^ pointed out that it is worthwhile to precisely determine the time interval between the scan session and the last attack as well.

It is widely accepted that stress is a potential trigger of migraine attacks^16^, but it has also been postulated that perturbations of the mesolimbic-cortical dopaminergic reward circuitry could contribute to migraine pain^17^. Pain and reward are seemingly opponent processes, but in reality there is a strong association between them, since pain and reward experiences are processed by many interacting and overlapping brain structures^18^. Numerous fMRI studies conducted in the last years have supported this observation. For instance, heightened activation of the nucleus accumbens (NAcc) and anterior cingulate cortex (ACC) were found following a noxious heat stimulus^19^ and enhanced activations were detected in the insular and orbitofrontal cortex (OFC) in response to pain-related words^20^. In addition, increased dopamine release was detected in the NAcc from the vental tegmental area (VTA) not only for primary rewards such as drugs or food^18^, but for aversive outcomes as well^21^. Vaajoki *et al*.^22^ found that pleasurable music decreased pain sensitivity^22^; and pain itself (especially chronic pain) was often accompanied by alterations in the brain reward circuitry^23^, increasing the possibility of avoidance behaviour and comorbid affective disorders^24,25^. Jin *et al*.^26^ reported similar results in migraine, stating that the structural and functional deficits of the OFC and the ACC could be essential in relating the negative affective components of migraine pain to later apathetic behaviour or depression.

Furthermore, the activity of the reward circuit could be considered as a biomarker of analgesic efficacy as well^23,27^. Felice *et al*.^28^ found that sumatriptan and CGRP_8–37_ treatment selectively elicited relief of ongoing cephalic but not non-cephalic pain, stimulated the dopamine efflux in NAcc and elicited conditioned place preference in rats. Based on these translational findings, the pain circuit is vulnerable to the signals of primary rewards, which have a direct positive value for the individual, since the stimulus, such as pain relief, is rewarding in itself^23,29^. However, it is still unclear whether these associations could be found in the case of secondary rewards as well, which do not have a direct immediate value and are not sensitive to saturation, therefore owns a relatively stable value (eg money)^30^. This question is especially interesting in cycling headache syndromes such as episodic migraine, where the pain and other somatic and behavioural symptoms are not constantly present^31^.

In addition, beyond the nature of rewards, it is worthwhile to investigate separately the neural responses during different stages of reward processing^32^. Although our understanding of the secondary reward anticipation and consumption in migraine is very limited, a recent study of fibromyalgia patients^33^ found reduced medial prefrontal cortex (mPFC), ACC and VTA activity during gain anticipation, and heightened mPFC activity during non-punishment outcomes compared to controls. Based on these differences in corticostriatal processing during reward anticipation and consumption in chronic pain, we could hypothesize differences in migraine as well.

For this reason, the aim of the present study is twofold: (1) explore the potential differences in brain response between migraine patients and non-headache healthy controls during reward/loss anticipation and consumption (2) investigate the potential influence of the duration of migraine history, attack frequency and the time of the last migraine attack before the scan session on the neural activations related to reward/loss processing.

## Results

### Behavioural results

A repeated measures ANOVA was conducted on the reaction times (RTs) to the target stimulus. In the total sample, surprisingly, the main effect of cue (reward and loss cues) showed only a trend toward significance (Greenhouse-Geisser correction: F(1.317, 89.588) = 3.182; p = 0.066; η^2^p = 0.045), as well as the block x cue interaction (Greenhouse-Geisser correction: F(1.572, 106.918) = 2.892; p = 0.072; η^2^p = 0.041). The main effect of block were not significant (Greenhouse-Geisser correction: F(1,68) = 0.078; p = 0.781; η^2^p = 0.001). These results could indicate that, comparing reward or loss cues to neutral cues, the responses were not quicker, and the RTs were unchanged as the task progressed. However, further analysis (paired t-tests) revealed significant differences between reward versus no-incentive cues (t = 3.908, df = 69, p < 0.001) and loss cues versus no-incentive cues (t = 3.095, df = 69, p < 0.001) in the first block, but did not show significant differences in the second block (t = 0.841, df = 69, p > 0.05; t = 0.080, df = 69, p > 0.05, respectively). From the means of RTs we could also conclude that, compared to the first block, in the second block participants did not give slower responses to reward or loss cues, but were quicker to the no-incentive cues (first block: M = 246.243, SD = 6.393; second block: M = 235.055, SD = 9.319) (main behavioural findings are shown in Supplementary Figs S1–S6). This acceleration could explain the non-significant results regarding cue x block interactions.

In addition, the main effect of group as between subject factor (F(1) = 0.339; p = 0.562; η^2^p = 0.005) and the cue x group interactions were not significant (Greenhouse-Geisser correction, F(1.317, 89.588) = 0.405; p = 0.582; η^2^p = 0.006), indicating no behavioural differences between migraine and control groups (for more information see Supplementary Table S1).

### Functional results

In the first step, the task related activations were tested in each group separately and our findings were in line with the results of previous studies^30,32,34–36^. The reward and loss anticipation contrasts showed several positive activations covering areas of thalamus and striatum, while many occipital and prefrontal areas yielded activations for reward and loss outcomes. Areas of OFC/vmPFC were also shown significant activity during the receipt of reward and losses (See Supplementary Tables S2–S5 and Figs S7, S8 for details). Our findings also seem to concord with the results of a recent meta-analysis^37^.

**Figure 1:**
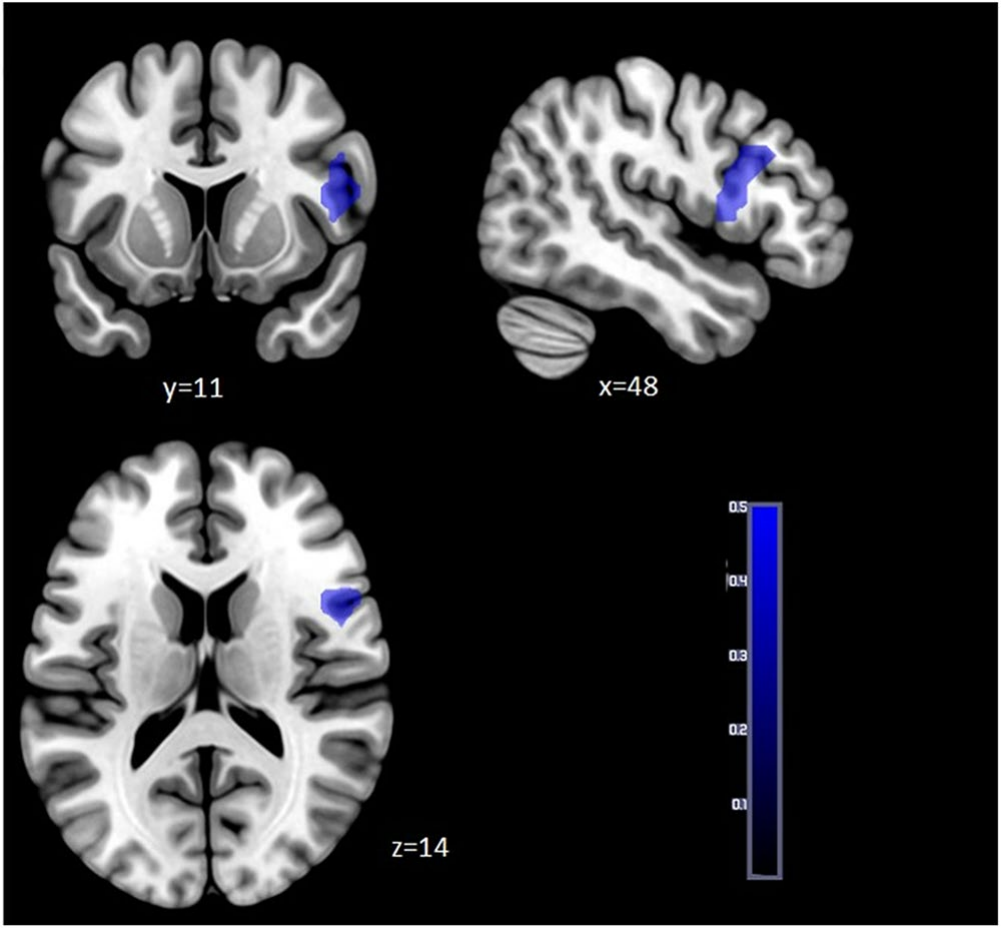
Group differences yielded for reward consumption. Neural activation (p_FWE_ < 0.05) in right inferior frontal gyrus pars opercularis (MNI coordinates: x = 48 y = 11 z = 14) for reward vs. neutral consumption contrast (success-neutral outcome) were decreased among migraineurs compared to healthy controls. Statistical maps were visualized on the MNI 152 template brain provided in MRIcroGL (https://www.mccauslandcenter.sc.edu/mricrogl/home).

In the second step, neural responses yielded to reward/loss anticipation and consumption in migraine and control groups were compared by two sample t-tests, controlling for the effects of age and sex. We did not detect any group differences in the anticipation phase of reward processing (win-neutral cue or loss-neutral cue contrasts), however, the analyses revealed significant group differences in the consumption phase, namely in the success-neutral outcome contrast (You won, No loss vs. No change) (for the possible outcomes see Table 1). Migraineurs, compared to controls, showed significantly decreased activations in one cluster: covering the right inferior frontal gyrus pars opercularis (p_FWE_ = 0.008, k = 130, MNI coordinates: x = 48 y = 11 z = 14) (see Fig. 1).

**Table 1:** Construction of the MID task.

	Success Index				
	Success		Fail		Neutral
Cue	+Ft	−Ft	+Ft	−Ft	0Ft
Visibility of the target	400 ms	400 ms	100 ms	100 ms	250 ms
Outcome	You won	No loss	No gain	You lost	No change

Note. Ft = the official abbreviation of the Hungarian currency.

After the group comparisons, separate analyses were conducted among migraineurs. To determine the influence of migraine attacks on the neural activations related to reward/loss processing, the duration of migraine history, attack frequency and the time of the last migraine attack before the scan session were included in the analyses as covariates of interest. All of the analyses were controlled for the effects of age and sex.

In light of the above, in the second step, we investigated the correlation between the BOLD response and the duration of migraine history and attack frequency, but we could not find any significant correlations between these clinical characteristics and brain activations yielded for reward/loss anticipation or consumption. However, we found interesting activation patterns when the time of the last attack before the scan session were entered in the analysis as covariate of interest (all scans were performed at least 48 hours after the last migraine attack).

**Figure 2:**
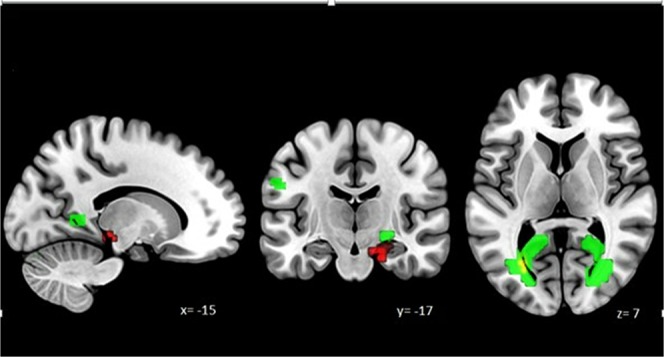
Overview of activated regions during loss processing in connection with the time of pre-scan attacks. Activations yielded for loss anticipation (loss-neutral cue- red) and loss consumption (loss-neutral outcome and failure-neutral outcome- green) contrasts in connection with the time of the last migraine attack *before* the scan session. Overlapping regions are presented in yellow. Statistical maps were visualized on the MNI 152 template brain provided in MRIcroGL (https://www.mccauslandcenter.sc.edu/mricrogl/home).

During *loss anticipation* (loss-neutral cue contrast), the activity in one cluster, covering the peaks of right parahippocampal gyrus and right hippocampus (see Table 2, Fig. 2), was negatively correlated with the time of pre-scan attacks. In other words, these areas were more active, when the time of the last attack was closer to the scan session. The *reward anticipation* contrast did not reveal significant activations in relation of the time of pre-scan attacks.

**Table 2:** Peak activations during loss anticipation in the relation of the time of pre-scan attacks (in hours) controlling for age and sex.

Contrast	Slope	Cluster size (voxel)	Peak T-value	Coordinates (MNI)			Region	Hemisphere
				x	y	z		
Loss-neutral cue	Negative	94	5.84	18	−19	−22	Parahippocampal gyrus	Right
			5.52	24	−25	−16		
			5.38	21	−22	−19		
			4.52	15	−10	−19	Hippocampus	Right

Note. Cluster-level p_FWE_ < 0.05.

In addition, during reward consumption (gain-neutral outcome and success-neutral outcome contrasts), increased activity was found in four clusters covering several regions such as the lingual gyrus, middle part of the occipital gyrus or the calcarine sulcus in relation to the time of pre-scan attacks. This means that these regions showed more activity as more time had passed between the last attack and scan session. Similarly activated regions were found during loss consumption as well (loss-neutral outcome and failure-neutral outcome) (see Table 3). Figure 2 shows discrepancies in the neural response yielded to loss anticipation and loss consumption, in connection with the time of pre-scan attacks, and Supplementary Fig. S9 illustrates the relationship of the neural activations and pre-scan times.

**Table 3:** Peak activations during reward/loss consumption in the relation of pre-scan attacks (in hours) controlling for age and sex.

Contrast	Slope	Cluster size (voxel)	Peak T-value	Coordinates (MNI)			Region	Hemisphere
				x	y	z		
Gain-neutral outcome	Positive	1016	7.21	−24	−67	11	Calcarine sulcus	Left
			6.94	−24	−70	5		
			6.81	−15	−49	5		
			6.67	−24	−61	8		
			5.73	3	−67	17		Right
			6.91	15	−79	17	Cuneus	Right
			5.69	6	−70	23		
			5.57	6	−70	23		
			5.65	12	−91	−7	Lingual gyrus	Right
			4.90	0	−70	2		Left
			5.55	−45	−61	−1	Middle temporal gyrus	Left
			4.83	−24	−94	11	Middle occipital gyrus	Left
		73	5.68	−30	−73	−22	Cerebelum_6	Left
			5.42	−39	−70	−22	Cerebelum_Crus1	Left
		207	5.42	36	−76	−16	Fusiform gyrus	Right
			5.41	27	−64	−16	Inferior temporal gyrus	Right
			4.74	42	−64	−10		
		132	5.39	18	−58	53	Superior parietal gyrus	Right
			5.37	9	−67	62	Preuneus	Right
			4.99	6	−52	65		
Success-neutral outcome	Positive	151	7.50	−27	−67	5	Calcarine sulcus	Left
			7.39	−15	−49	5		
		450	5.89	12	−79	17	Cuneus	Right
			4.33	6	−70	23		
			3.54	3	−88	14		Left
			4.98	3	−67	14	Calcarine	Right
			4.84	27	−52	5		
			4.72	18	−52	5		
			4.36	−9	−79	14		Left
			4.31	3	−70	−1	Lingual gyrus	Right
			3.80	12	−73	−10		
		60	4.54	24	−76	−13	Fusiform gyrus	Right
			3.75	33	−79	−16		
			3.88	24	−67	−16	Cerebelum_6	Right
			3.84	39	−73	−13	Inferior occipital gyrus	Right
Loss-neutral outcome	Positive	177	4.83	27	−55	5	Calcarine sulcus	Right
			4.28	18	−76	14		
			4.11	15	−82	14		
			4.39	12	−85	17	Cuneus	Right
			4.33	3	−67	20		
			3.84	9	−82	26		
			3.87	18	−55	2	Lingual gyrus	Right
		113	5.85	−27	−61	5	Calcarine sulcus	Left
			5.68	−18	−52	5		
Failure-neutral outcome	Positive	139	6.94	−27	−61	5	Calcarine sulcus	Left
			6.70	−27	−67	5		
			6.43	−18	−52	5		
		164	5.84	27	−55	5	Calcarine sulcus	Right
			4.41	18	−52	5		
			4.12	18	−76	17	Cuneus	Right
			3.72	9	−82	26		
			3.72	12	−76	20		
			3.91	42	−73	14	Middle occipital gyrus	Right

Note. Cluster-level p_FWE_ < 0.05.

Our results show that the behavioural findings were unrelated to time since the last migraine attack (Spearman’s rho were ranging between −0.043 and 0.250; p > 0.05). Significant associations were exclusively found between the BOLD responses and times of pre-scan attacks.

## Discussion

In this study, neural responses of patients with migraine without aura were compared to pain-free healthy controls during monetary reward/loss anticipation and consumption. We found altered neural processing in migraineurs in reward consumption (success-neutral outcome), but not during anticipation of either rewards or losses. We also analysed the association between BOLD response and three migraine characteristics, such as the attack frequency per month, duration of migraine history (in years) and time (in hours) of the last attack before the scan session. Although the attack frequency and duration of migraine were unrelated to reward and loss processing in our study, the time of the last attack pre-scan had a significant impact on loss anticipation and reward/loss consumption.

### Reduced VLPFC activity to monetary reward consumption in migraine

Accumulated evidence suggest^18,38^ that the mesolimbic cortical reward circuitry may play an important role in migraine pain. Rewarding stimuli such as different drugs^28,39^ or relief from acute pain could decrease pain sensitivity^23,27^, and dysfunctions of this circuitry could contribute to the maintenance of chronic pain^18^. Previous studies found reduced BOLD activity of the ACC and insular cortex^8^, and structural deficits in the OFC among migraine patients^26^. These functional and structural perturbations were almost exclusively found in chronic migraine where the pain experience is (almost) constant^40,41^; or in connection with primary rewards where the stimulus itself is rewarding such as pain relief^17,23^. In our study we explored the impact of (secondary) monetary rewards/losses on neural activity in episodic migraine and we found decreased activation in one cluster covering the right inferior frontal gyrus (rIFG) pars opercularis during reward/no loss consumption (success-neutral outcome) in migraineurs compared to healthy controls.

The IFG along with the anterior insula is considered to form the ventral corticolimbic control pathway^42^. This reactive system was adapted to control behaviour and cognition in low-predictable environments where previously formed context models cannot be used. Instead, behaviour is led by momentary sensory stimuli which can be negative (“I need to get away from danger”) or positive (“I have to catch the reward before it gets away”)^43^. Hence, the reactive ventral corticolimbic pathway “relates stimuli to the experienced self in the here and now”^42^. Our results seem to converge with this hypothesis, since we detected decreased IFG activation in migraine patients during a task which provide a more or less uncertain environment. It is worthwhile to mention, that generally more uncertainty is present in anticipation than in consumption phase of reward processing, however a certain level of uncertainty is detectable during consumption as well, which could be derived from the mismatch between the predicted reward or punishment and that actually delivered. The mismatch can lead to positive or negative prediction errors (when the outcome is better or worse than previously expected), which could create a more or less predictable environment for the individual^30^. Previous studies also reported the involvement of IFG in detection of prediction errors^44^, especially during the receipt of reward/punishment^45^. The MID paradigm requires narrow spatial and temporal focus where behaviour is guided reactively by the actual stimuli, instead of previous context models. According to our results, this reactive attentional system is negatively affected in episodic migraine, especially during reward consumption.

The theoretical model of Tops and Boksem^43^ were empirically tested by subsequent studies too, which have also found that IFG or more broadly the ventrolateral prefrontal cortex (VLPFC) is sensitive to saliency, attentional load and stimulus frequency and it shows more activity in individuals with higher reward sensitivity^46,47^. Cho *et al*.^48^ also detected increased VLPFC activity in connection with higher reward sensitivity. In their study, reward sensitivity was conceptualized as consummatory pleasure derived from the joy in response to a delectable cue (ie “The smell of freshly cut grass is enjoyable to me”)^48,49^. Moreover, previous findings suggested the important role of lPFC in the prediction of reward information^50^ and in stimulus-reward associations as well^51^. Dillon *et al*.^32^ also detected increased IFG activation during the receipt of monetary rewards, enhancing that this area was specifically involved in reward but not loss consumption.

Based on the above, our results could indicate decreased immediate reactivity to the receipt of monetary rewards in episodic migraine suggesting a slower associative learning between sensory processing and target-triggered responses. It is also presumable, that these deficits are only linked to the consumption of rewards but not to punishments/losses.

Nevertheless, it is possible that the main difference in the reward processing of migraineurs and controls is not in the consumption of “net gains” but in the perception or interpretation of “no loss” outcomes. The base of this assumption is the interesting finding that we only detected reduced VLPFC activity in the success-neutral outcome contrast, where success was modelled by two feedbacks, namely the “you won” and the “no loss” outcomes. However, no group difference was observed during win-neutral outcome (You won vs. No change), where the “no loss” outcome was not included in the contrast. One possible explanation for this result could be that, for migraine patients, signals of no loss are not as rewarding as the signals of net gains.

In order to support this assumption in a post-hoc analysis we tested the group differences for the contrast of no loss versus neutral outcome, as well. The group comparison revealed that migraineurs, compared to controls, showed significantly decreased activations in one cluster (p_FWE_ = 0.011, 123 voxels, T = 3.96) covering the peaks of left fusiform gyrus (MNI: x = −36 y = −64 z = −13), left cerebellum_6 (MNI: x = −36 y = −70 z = −22) and left cerebellum_Crus1 (MNI: x = −39 y = −70 z = −22). Although different areas were activated for this contrast than for success-neutral outcome contrast, the results congruently indicate a neural hypoactivity of migraineurs compared to controls during reward consumption. Our results seem to be in line with previous human and rodent studies as well, highlighting the role of cerebellum in the processing of emotional stimuli and in cognition^52^ and in reward omission and reward anticipation^53^.

In addition, since we found similar BOLD responses yielded for loss outcomes and reward/loss cues between migraineurs and controls, we could assume intact loss consumption and reward/loss anticipation processes in episodic migraine.

It remains unclear however, whether the neural activation during reward consumption in migraine could be similar to the brain response found in different chronic pain syndromes. A recent study for example, detected different BOLD-responses to reward consumption between fibromyalgia (FM) patients and controls^33^. The authors found increased mPFC activity yielded for no loss outcomes compared to controls, which could indicate that FM patients tend to process no loss outcomes as a reward or relief rather than just a “zero sum” situation like healthy controls. Although these findings seem to contradict our results, we should take into consideration that (1) FM is a chronic pain condition unlike episodic migraine and (2) in the study of Martucci *et al*.^33^ patients were taking various drugs (such as NSAID, SSRI, SNRI etc.) to alleviate pain and stabilize their mood which may influence these results. In spite of these results our interpretation of secondary reward processing in episodic migraine remains limited, and it needs to be confirmed with additional studies.

### Brain activity during reward/loss processing in relation to migraine characteristics

Neuroimaging studies identified various structural and functional alterations in the migraine brain not just during attacks or pre-/post-ictal periods, but interictally, as well^54–56^. These changes were positively correlated with the attack frequency and duration of migraine history^13,57^, indicating a potential “scar effect” of migraine attacks. However, due to the lack of longitudinal studies, it is difficult to determine if these amendments are the results of the attacks, or they may predispose individuals to have migraine^9^. According to this, we were interested in the potential influence of these clinical characteristics on the neural response during reward processing. Against expectations, we did not detect significant correlations between attack frequency^14^, duration of migraine^13^ and BOLD response to reward/loss anticipation and consumption. Apparently, these characteristics do not affect reward processing in episodic migraine, however we need to note that most of our sample consisted of low frequency migraineurs (less than 3 attacks per month), which could also contribute to the non-significant results.

In addition to duration and frequency of migraine, Schwedt *et al*.^15^ found that it is worthwhile to explore the proximity of the attacks as well. They detected significant positive correlations between pain tolerance thresholds at the head and forearm and the number of hours until the next migraine headache in interictal stages. We found negative correlation between the time of the last migraine attack before the scan session and the neural activation yielded to loss anticipation, and we detected positive correlation between the time of pre-scan attacks and BOLD response during reward/loss consumption.

During loss anticipation (loss-neutral cue contrast) one cluster covering the peaks of right parahippocampal gyrus and right hippocampus (for details see Table 2) showed increased activity when the time of the last attack was closer to the scan session. Hippocampus is known to be involved in various processes such as memory consolidation^58^ and organization^59^; stress response^60^; context specific behavioural inhibition and aversive conditioning^61^, or anticipation and novelty encoding^62^. In addition, recent studies pointed out its role in pain processing as well, detecting increased activation yielded to anticipated threatening painful events^63,64^; volumetric and functional differences in high-frequency versus low frequency migraineurs in response to painful heat stimulation^14^; and heightened activity to anxiety induced hyperalgesia^61^. These pain related studies concluded that the hippocampal formation tends to amplify aversive events preparing the organism (and its behaviour) for the worst possible outcome. Our results are in line with these conclusions, as hippocampus and parahippocampal gyrus showed increased activity during the expectancy of an aversive but not the rewarding cues. Furthermore, our findings also add to the existing knowledge, since in contrast with most of the previous studies where neural and/or psychological changes attributed to the migraine attack are typically detected within the last 48 or 72 hours prior to the headache^4,7,65^, we found a gradually enhanced hippocampal activity in relation to the time of the attacks, even before this period.

In addition, we also found positive correlation between the time of pre-scan attacks and neural response to *reward/loss consumption* (see Table 3). We observed a relative increase in the activity of visual areas (eg calcarine, cuneus, lingual gyrus) as more time had passed between the last migraine attack and the scan session. Our findings seem to converge with the results of Vincent *et al*.^12^ who found enhanced interictal reactivity of the visual cortex among migraineurs. One could also argue that these areas showed less neural activity near the migraine attacks compared to headache free intervals.

Furthermore, since we found very similar BOLD responses yielded for both reward and loss outcomes in relation to the time of the last migraine attack, we could assume that processes that are common to receipt of rewards and losses are equally affected near the attacks. Further studies are definitely needed to elucidate these associations.

### Limitations

Like most studies, ours has some limitations that should be noted. Although we measured behavioural responses in connection with the MID task and from the results we could conclude that our participants were motivated, we did not assess explicitly the perceived probability of reward, or participants’ motivation to win or avoid loss. It would have also been good to measure reward sensitivity of participants and include it in the analysis. Furthermore, mostly low-frequency migraineurs participated in our study; therefore, we could not properly measure the impact of high-frequency attacks on reward processing.

Finally, since we investigated a relatively new field by using the MID task in episodic migraine, it remains to be seen if the current findings could be replicated by further studies.

## Conclusion

To the best of our knowledge, this is the first study investigating monetary reward and loss processing in episodic migraine. Our data suggest intact reward and loss anticipation but significantly altered reward consumption in migraine, compared to controls, indicating a decreased reactivity to (sometimes low predictable) monetary rewards. Following the distinction of Berridge *et al*.^66^, we also could conclude that neural activations in episodic migraine show deficits only during the later phases of reward processing, namely in the hedonic or “liking” phase; while neural responses during the motivational or “wanting” phase are similar to headache free controls.

In addition, our results could also raise the possibility that neural responses yielded for loss anticipation and reward/loss consumption could be altered by the proximity of the last migraine attack far beyond peri-ictal stages. Although our findings shed light to the interesting association between anticipation processes towards aversive events and migraine attacks, further prospective studies are needed to determine the causal relation between them.

Our results could contribute to the existing knowledge, highlighting that secondary reward processing is sensitive to the different characteristics of migraine state, not just ictally but also interictally, although it is unclear whether these interictal functional brain alterations interact with different pain related processes. However, these associations deserve increased attention and further investigations, in order to find and improve treatment options.

## Methods

### Participants

Participants, aged between 18–39 years, were recruited at a local neurological clinic, via general practices, and via university and newspaper advertisements. All volunteers were tested for eligibility.

Migraine without aura diagnosis was established by senior neurologists according to the International Classification of Headache Disorders third edition criteria (Headache Classification Committee of the IHS, 2013). Participants were included if they had at least one migraine attack per month, did not have chronic migraine (more than 15 attacks per month at least for 3 months) or aura symptoms and did not overuse headache medication. According to the often used protocol^15,67,68^, migraineurs refrained from using prophylactic medication for 3 months and migraine attack medication 48 hours prior the scan sessions. fMRI scans were performed at least 48 hours after the last attack.

Non-headache healthy controls were also examined by senior neurologists and were free from any type of primary or secondary headache, and they did not use any medication (oral contraceptives were not exclusion criteria). General inclusion criteria were right handedness assessed with a standardized handedness questionnaire^69^ and normal or corrected to normal vision. General exclusion criteria were any MRI contraindications, and any history of medical, neurological (except migraine for patients’ group) or psychiatric disorder diagnosed by senior neurologist and psychiatrist researchers.

Out of the 124 volunteers, 37 individuals did not meet the inclusion criteria (eg they had aura symptoms, hypertension, diabetes etc). Altogether 87 participants (38 migraine patients and 49 healthy controls) were included, however further 17 participants (9 migraineurs and 8 controls) were excluded from the analysis due to excessive movement, low quality of images and due to missing data derived from technical problems. The final sample comprised of twenty-nine patients with migraine without aura (23 females, mean age = 26.72; SD = 4.93) and forty-one non-headache healthy controls (24 females, mean age = 26.19; SD = 4.19).

All participants provided written informed consent before entering the study. The study was approved by the Scientific and Research Ethics Committee of the Medical Research Council (Hungary), and the work was conducted in accordance with the Declaration of Helsinki.

### Migraine symptoms and severity measures

All participants with migraine kept a headache diary. The time, duration, and strength of the attacks were recorded (see Table 4 for details) as well as the accompanying symptoms (eg photo-, phonophobia, nausea etc), and medication use.

**Table 4:** Demographic and clinical characteristics of migraine patients and healthy controls.

	Sex ratio (female:male)	Age	Duration of migraine history (years)	Attack frequency per month	Pre-scan attacks (hours)	Estimated total number of attacks
Migraine patients	23:6	26.72 ± 4.93	11 ± 7.53	3.64 ± 2.99	235.93 ± 263.95	392.8 ± 571.35
	N = 29	N = 29	N = 29	N = 29	N = 29	N = 25
Healthy controls	24:17	26.19 ± 4.19	NA	NA	NA	NA
	N = 41	N = 41				

Note. Data are expressed as means ± SD (standard deviation); NA = not applicable.

### fMRI paradigm

**Figure 3:**
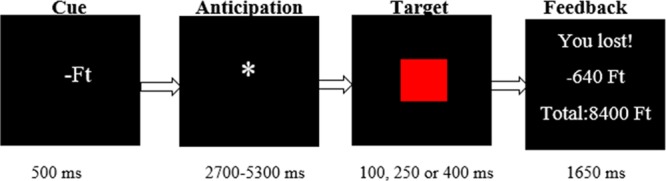
Design of the Monetary Incentive Delay (MID) Task. Note. Ft = the official abbreviation of the Hungarian currency.

Reward and punishment anticipation and consumption were modelled by a variant of the Monetary Incentive Delay (MID) Task^32,70,71^. At the beginning, participants were told that during this task they could gain money or avoid monetary loss, if they responded to the target (a red square) fast enough. Before the target appeared on the screen, a visual cue showed up (for 500 milliseconds), indicating a potentially rewarding outcome (+Ft - which is the official abbreviation of the Hungarian currency), losing (−Ft), or neutral (0 Ft) outcome. The cue presentation was followed by a variable time interval delay (ISI = 2700–5300 ms) while a white star was presented to the subjects. After this anticipation phase, the red target square appeared (for 100, 200 or 450 milliseconds), whereupon the subjects had to respond by pressing a button, as quickly as possible. Following the response, participants instantly got a feedback (for 1650 ms) which informed them (1) whether they won or lost money (2) how much they earned or lost during the trial (3) the total financial balance. The task contained 90 trials organized in two blocks. The trials were separated by a varying inter-trial interval (ranging from 1150–4050 ms). One trial lasted 9000 ms in total (see Fig. 3).

Participants were not aware of the fact that their success in the game were independent from their reaction times, since every trial was previously programmed to be successful or unsuccessful. Naturally, there were some circumstances that could overwrite the original script, for example participants could lose money in successful trials too, if they did not respond at all. In order to increase credibility, in successful trials the cues were visible for 400 ms, but in unsuccessful trials the cues were only detectable for 100 ms and in no change condition for 250 ms (for the construction of the task see Table 1). The order of conditions was pseudo-randomized. In addition, during data analysis, reaction times (RT) below 100 ms and above 1000 ms were considered invalid and were filtered out from the analysis.

Every participant previously practiced the task in a laptop, outside the scanner. To maximize engagement, we offered them to take part in a prize draw after the experiment, where they could win the money they collected during the MID task (~28€–65€).

### Functional data acquisition

In order to minimize the effects of circadian rhythm, all of the subject were scanned afternoon (after 3:00 p.m.). Participants were also asked to refrain from eating, smoking or consuming caffeine 4 hours before the examination.

fMRI scan was performed on a 3 T MRI scanner (Achieva 3 T, Philips Medical System). T2*-weighted images were obtained using echo-planar imaging sensitive to BOLD contrast (TR = 2500 ms, TE = 30 ms, FOV = 240 × 240 mm, in-plane voxel size = 3 × 3 mm; slice thickness = 3 mm). A series of high-resolution anatomical images were also acquired during the functional imaging session using a T1-weighted 3D TFE sequence with 1 × 1 × 1 mm resolution.

### Functional data analysis

Functional imaging data were analysed with SPM (Statistical Parametric Mapping) 12 software package (http://www.fil.ion.ucl.ac.uk/spm/software/spm12/ ^72^, implemented in MATLAB 2015b (MathWorks, Natick, MA, USA). After converting the raw data to NIfTI format, data were pre-processed following a standard sequence. The realigned functional images and mean images were co-registered to the structural image, then these structural images were segmented. Functional images were normalized in Montreal Neurological Institute (MNI) space and were spatially smoothed with an 8 mm full-width-at-half-maximum (FWHM) Gaussian kernel.

Pre-processed data were further checked using Artifact Detection Tools (ART; http://www.nitrc.org/projects/artifact_detect/ Whitfield-Gabrieli and Mozes, 2009, MIT) to identify linear and rotational head motion parameters and outliers in the global mean image time series, using a threshold of global signal >3 SD and motion >1 mm. The motion outliers were used as nuisance regressors in the first level models. In the last step of pre-processing, two independent researchers visually inspected the quality of the images.

First level analysis and group comparisons were performed in the framework of the general linear model (GLM) focusing to the BOLD (blood-oxygen-level-dependent) hemodynamic responses, yielded for reward/punishment anticipation and consumption. The task was modelled in event-related design, since the three types of incentive cues (win, loss, neutral) and the five types of feedback (you won, you lost, no gain, no loss, no change) were modelled with event-specific regressors.

For each individual GLM, six contrasts were defined, from which two represented the anticipation and four the consumption phase of reward/punishment processing. Neural responses during anticipation were assessed by win-neutral cue (+Ft vs. 0 Ft); and loss-neutral cue (−Ft vs. 0 Ft) contrasts. Activations yielded for consumption were measured by win-neutral outcome (You won vs. No change), loss-neutral outcome (You lost vs. No change), success-neutral outcome (You won, No loss vs. No change), and failure-neutral outcome (You lost, No gain vs. No change) contrasts, using one-sample t tests (for the construction of the task see Table 1). Regarding the activations of reward and loss anticipation/consumption, a whole brain analysis was carried out at an initial threshold of p < 0.001 uncorrected level with a cluster size >10.

In second-level analysis whole brain two sample t-tests were conducted to compare activations between the control and migraine group, while the effects of age and sex were controlled for. To adjust for multiple comparisons, cluster level family wise error corrected p_FWE_ < 0.05 values were reported as significant.

Following the group comparisons, a separate analysis was conducted in the migraine group, where correlations between task-related activations and the attack frequency per month, duration of migraine history (in years), and time (in hours) of the last attack before the scan session were investigated. Again, results were thresholded at *p*_FWE_ < 0.05 cluster-level using an initial threshold of *p* < 0.001 uncorrected, with a voxel size >10.

Activated clusters were identified using Automated Anatomical Labeling atlas (aal; Tzourio-Mazoyer *et al*., 2002) implemented in WFU Pickatlas toolbox^73–75^. Statistical maps were visualized on the MNI 152 template brain provided in MRIcroGL (https://www.mccauslandcenter.sc.edu/mricrogl/home)^76^.

### Statistical analysis

Behavioural data, such as reaction times in response to the target, were recorded by E-prime 2.0 software (Psychology Software Tools, Pittsburgh, PA), and were analysed using SPSS version 25 (IBM SPSS, IBM Corp, Armonk, NY), along with basic demographic and migraine symptoms related data. In functional data, one-sample-, and two-sample t-tests were performed, while behavioural and demographic data were analysed by paired t-tests and repeated measures ANOVA. All statistical tests were two-tailed with an alpha level of p < 0.05.

### Ethics statements

This study was approved by the Scientific and Research Ethics Committee of the Medical Research Council (Hungary). All participants provided written informed consent before entering the study and the work was conducted in accordance with the Declaration of Helsinki.

## Supplementary information

Supplementary Information

## Data Availability

The main fMRI contrast maps are available at the Open Science Framework repository, https://osf.io/f9udz.
