# Supplementary material for: Altered neural activity to monetary reward/loss processing in episodic migraine
Source: Sci Rep. 2019 Apr 1;9:5420. doi: 10.1038/s41598-019-41867-x (PMC6443660; doi:10.1038/s41598-019-41867-x)
Supplement: Supplementary file 1 — Supplementary Information [file 41598_2019_41867_MOESM1_ESM.pdf]

Supplementary Information

Altered neural activity to monetary reward/loss processing in episodic migraine

Natália Kocsel[1,2,3,4], Attila Galambos[1,2,5], Edina Szabó[1,2,5], Andrea Edit Édes[3,4], Máté Magyar[6], Terézia Zsombók[6], Dorottya Pap[4], Lajos Rudolf Kozák[7], György Bagdy[4,5], Gyöngyi Kökönyei[2,3,4] , Gabriella Juhász[3,4,8]

[1] Doctoral School of Psychology, ELTE Eötvös Loránd University, Budapest, Hungary
[2] Institute of Psychology, ELTE Eötvös Loránd University, Budapest, Hungary
[3]SE-NAP2 Genetic Brain Imaging Migraine Research Group, Semmelweis University, Budapest, Hungary
[4]Department of Pharmacodynamics, Faculty of Pharmacy, Semmelweis University, Budapest, Hungary
[5]MTA-SE Neuropsychopharmacology and Neurochemistry Research Group, Hungarian Academy of Sciences, Semmelweis University, Budapest, Hungary
[6]Department of Neurology, Faculty of Medicine, Semmelweis University, Budapest, Hungary
[7] MR Research Center, Semmelweis University, Budapest, Hungary
[8]Neuroscience and Psychiatry Unit, The University of Manchester and Manchester Academic Health Sciences Centre, Manchester, United Kingdom

*** Correspondence:**
Gyöngyi Kökönyei
kokonyei.gyongyi@ppk.elte.hu

Supplementary Table S1. Summary of means, standard deviations and group differences of reaction times

Condition	Migraine M (SD) in milliseconds	Control M (SD) in milliseconds	t	df	p
Block 1					
Win cue	225.052 (36.617)	229.088 (40.134)	-0.430	68	0.669
Loss cue	228.206 (40.957)	233.357 (34.480)	-.0569	68	0.571
Neutral cue	240.528 (55.674)	251.957 (50.503)	-0.894	68	0.375
Block 2					
Win cue	227.760 (63.827)	229.890 (39.145)	-0.160	68	0.874
Loss cue	236.709 (44.306)	236.540 (41.019)	0.016	68	0.987
Neutral cue	230.139 (86.223)	239.971 (69.469)	-0.528	68	0.600
N	29	41			

Note. SD= standard deviation

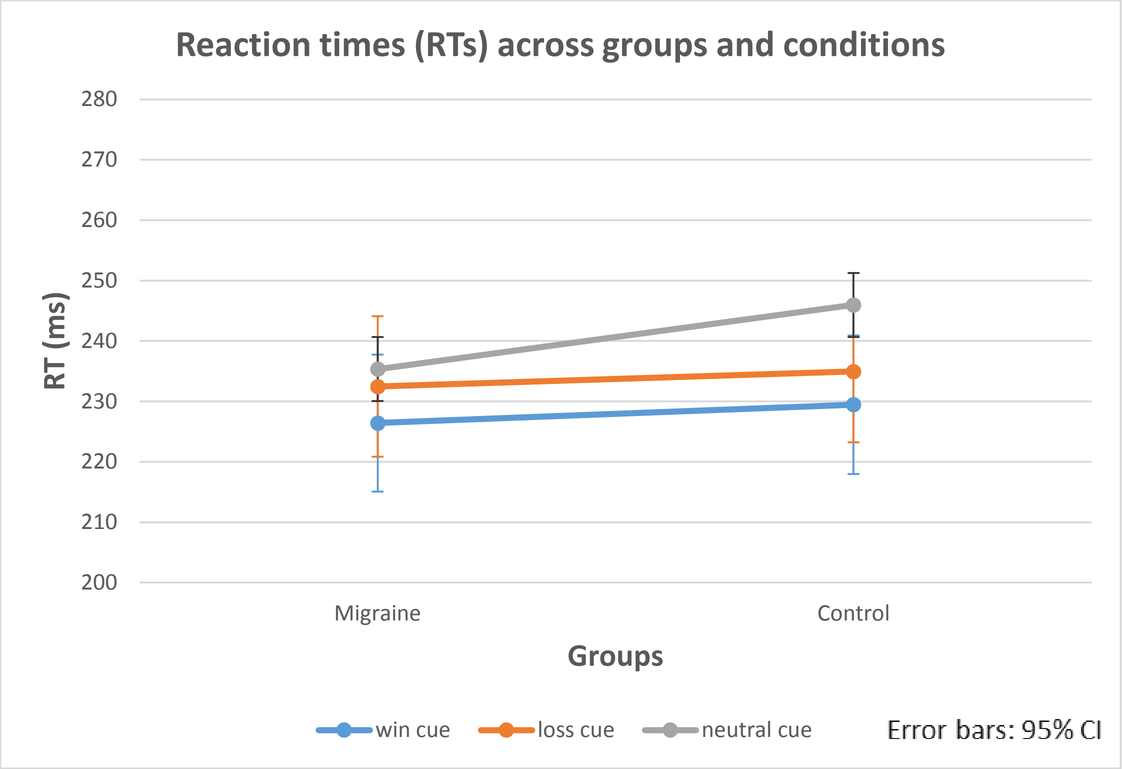

Supplementary Figure S1. Overall reaction times across conditions and groups
Note. The figure illustrates the main reaction times across groups and conditions. The difference between migraine and control groups is statistically non-significant. For exact results see Table S1 above.

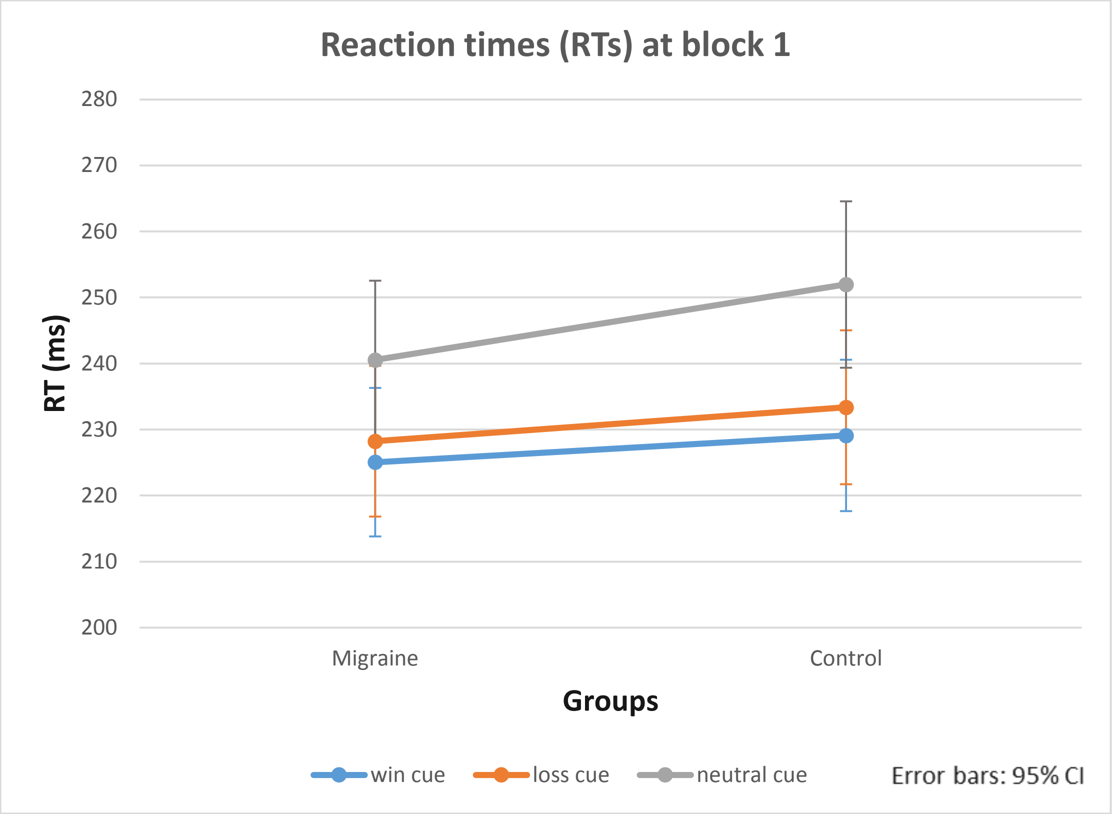

Supplementary Figure S2. Reaction times across conditions and groups in block 1.
Note. The figure illustrates the main reaction times across groups and conditions. The difference between migraine and control groups is statistically non-significant. For exact results see Table S1 above.

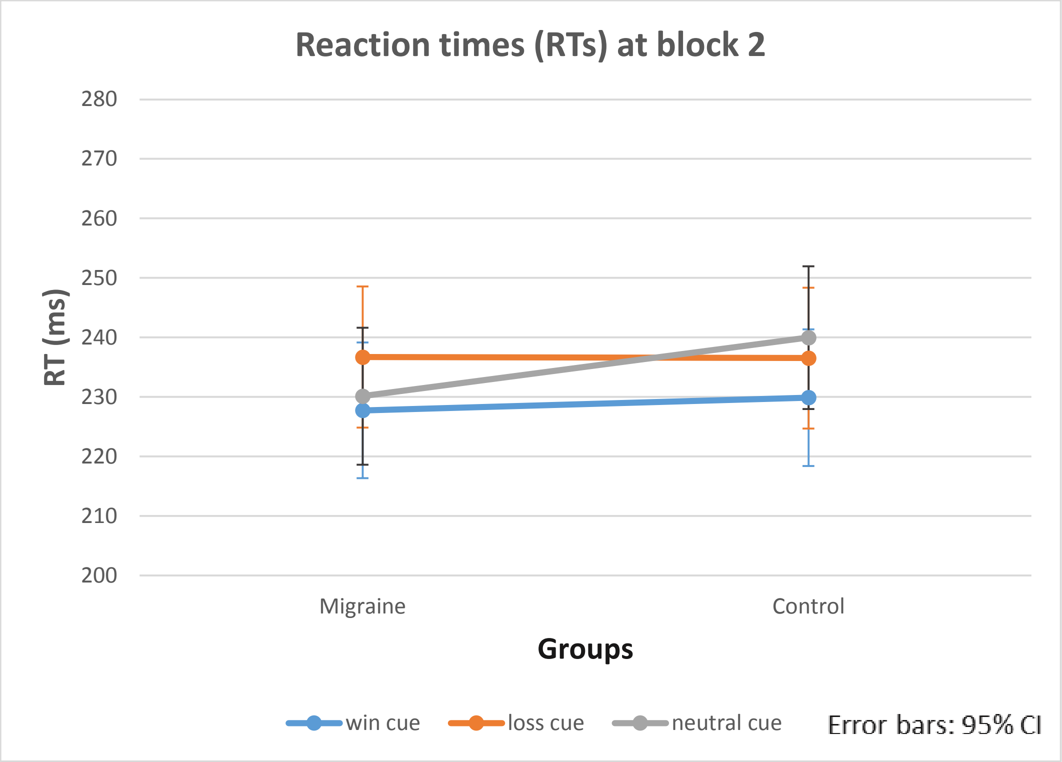

Supplementary Figure S3. Reaction times across conditions and groups in block 2.

Note. The figure illustrates the main reaction times across groups and conditions. The difference between migraine and control groups is statistically non-significant. For exact results see Table S1 above.

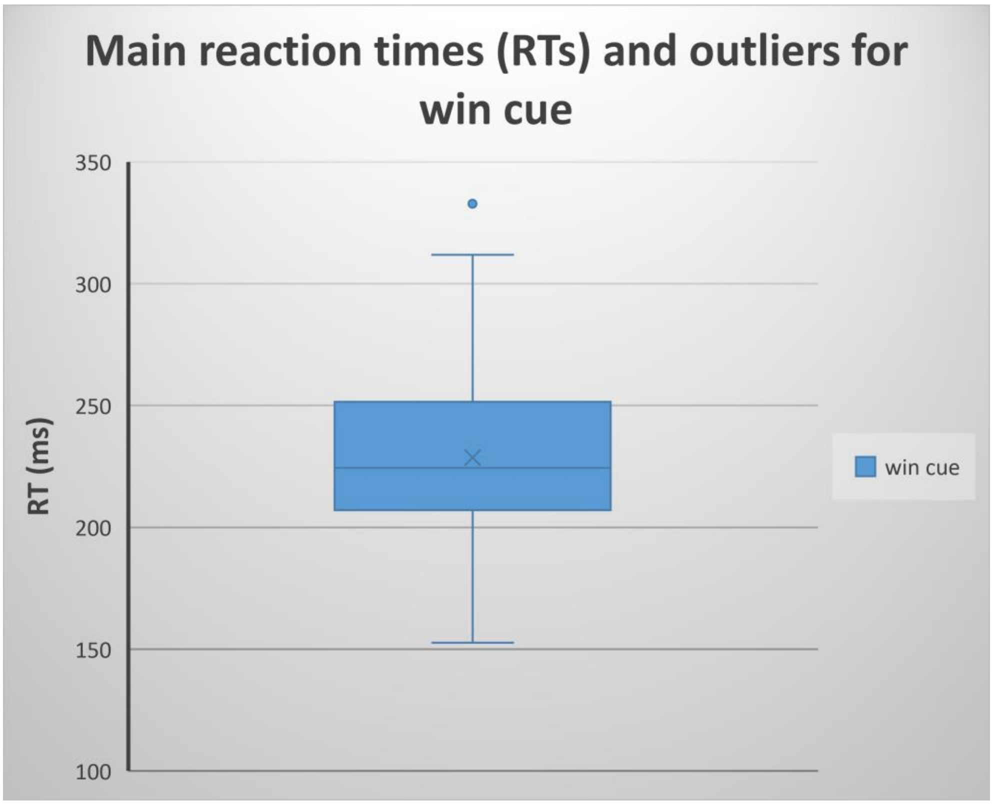

Supplementary Figure S4. Reaction times and outliers for win cue.

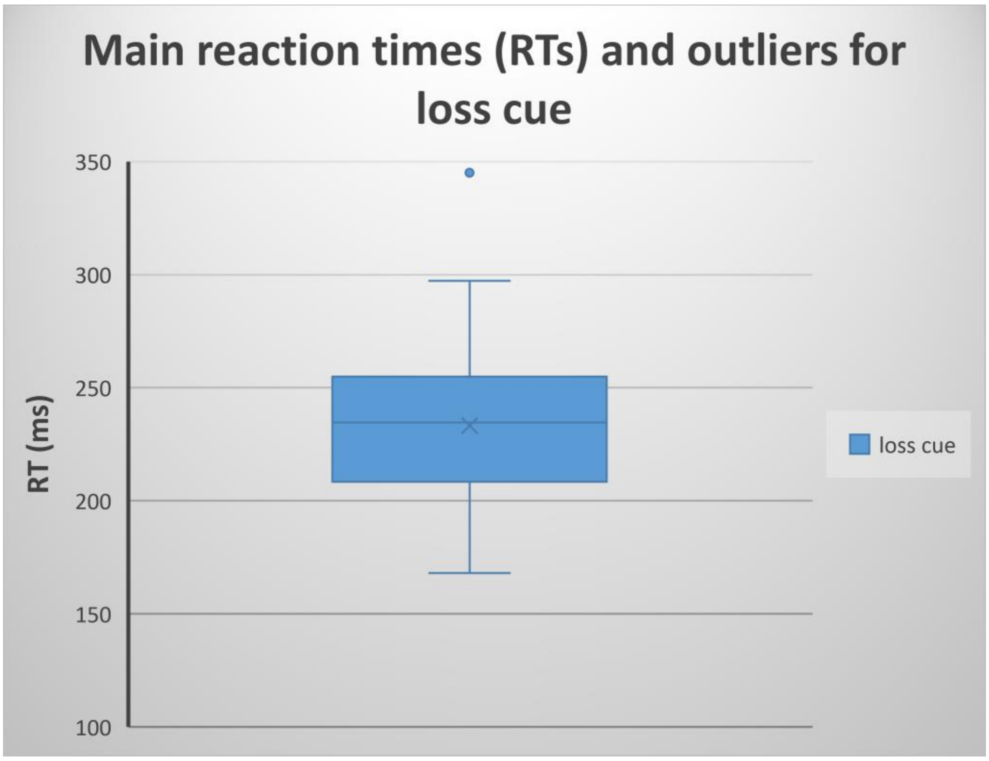

Supplementary Figure S5. Reaction times and outliers for loss cue.

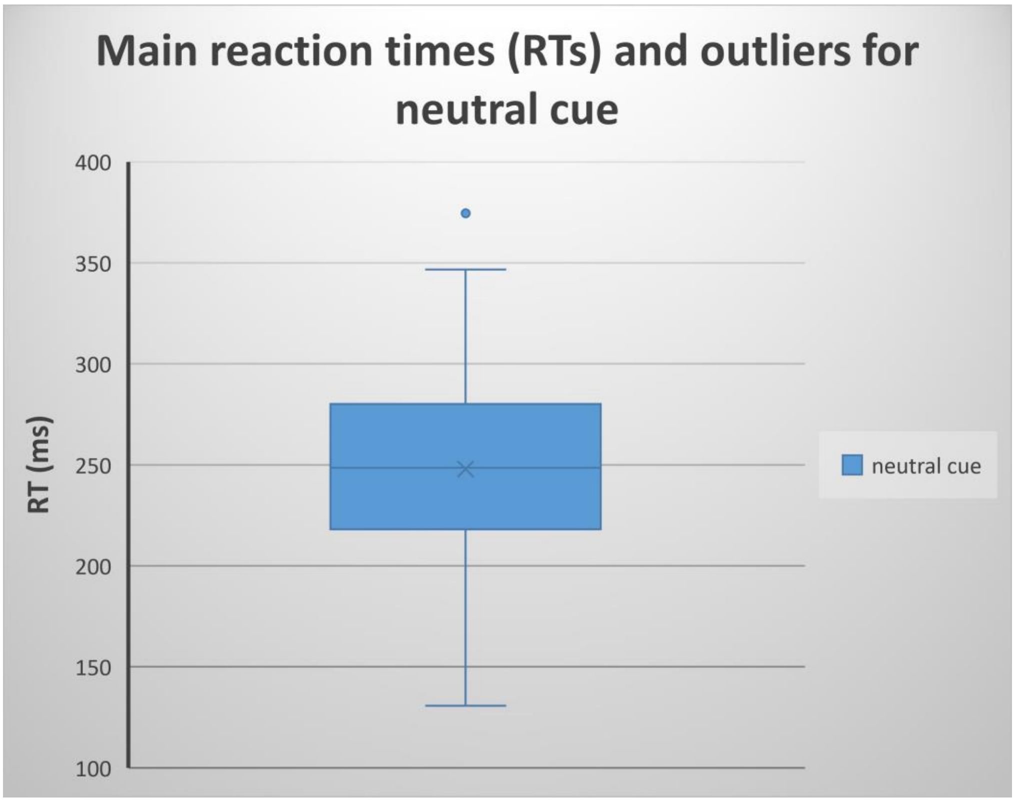

Supplementary Figure S6. Reaction times and outliers for neutral cue.

Supplementary Table S2. Regions activated in migraine/control groups during reward anticipation

Contrast	Groups	Cluster size (voxel)	Peak T-value	Coordinates (MNI) x	y	z	Region	Hemisphere
Win-neutral cue	Migraine	366	5.89	-15	-91	-4	Middle occipital gyrus	Left
			4.83	-24	-94	11		
			4.70	-24	-88	17		
			3.89	-33	-82	-7	Inferior occipital gyrus	Left
			3.67	-36	-85	-10		
		303	5.59	18	-88	-1	Calcarine sulcus	Right
			5.15	27	-88	5	Middle occipital gyrus	Right
		155	5.23	-6	-10	-1	Thalamus	Left
			3.79	6	-13	-1	Thalamus	Right
			3.68	18	-13	11		
	Control	198	5.74	21	-82	20	Superior occipital gyrus	Right
			5.34	18	-79	29		
			4.09	21	-70	20	Cuneus	Right
		86	5.20	48	-1	50	Precentral gyrus	Right
			4.77	42	-7	53	Middle frontal gyrus	Right
		83	5.18	24	-91	-4	Lingual gyrus	Right
			4.07	36	-88	-4	Inferior occipital gyrus	Right
			3.95	45	-79	-1	Middle occipital gyrus	Right
			3.47	42	-82	2		
		158	5.17	-6	-13	-11	Thalamus	Left
			4.81	-3	-16	-1		
		249	4.87	-24	-82	23	Middle occipital gyrus	Left
		85	4.72	-24	-91	-10	Inferior occipital gyrus	Left
			4.35	-30	-91	-10		
			3.84	-21	-94	2	Middle occipital gyrus	Left
			3.42	-30	-88	2		

	104	4.43	18	-1	17	Caudate	Right
		4.32	12	-1	17		
		3.61	9	11	2		
		3.44	9	14	-7		
	88	4.18	0	-1	56	Supplementer motor area	Left
		4.13	-3	2	68		
		3.71	-3	5	62		

Note. Cluster-level $p_{FWE}<0.05$

Supplementary Table S3. Regions activated in migraine/control groups during loss anticipation

Contrast	Groups	Cluster size (voxel)	Peak T-value	Coordinates (MNI) x	y	z	Region	Hemisphere
Loss-neutral cue	Migraine	1114	6.91	-15	-91	-4	Middle occipital gyrus	Left
			6.73	-18	-88	-7		
			6.06	-27	-91	8		
			5.90	-21	-91	8		
			5.90	-24	-94	11		
			4.95	-39	-85	5		
			6.06	36	-85	8		Right
			6.82	18	-88	-4	Lingual gyrus	Left
			4.78	-33	-82	-4	Inferior occipital gyrus	Left
			4.19	24	-79	23	Superior occipital gyrus	Right
			4.11	45	-79	-4	Inferior occipital gyrus	Right
			3.96	-6	-79	-16	Cerebelum_6	Left
		339	4.98	-6	-16	2	Thalamus	Left
			4.78	6	-16	11	Thalamus	Right
			4.26	6	-13	2		
			4.40				Caudate	Left
		104	4.96	-3	5	65	Supplementer motor area	Left
			4.72	-6	-10	56		
		232	4.89	27	-55	-28	Cerebelum_6	Left
			4.74	21	-52	-25		
			4.53	3	-58	-22	Vermis_6	
			4.10	15	-64	-37	Cerebelum_8	Right
	Control	139	4.89	-9	-10	17	Thalamus	Left
			4.23	-6	-13	5		
			3.73	-12	2	-4	Pallidum	Left
		115	4.55	12	-1	17	Caudate	Right

Note. Cluster-level $p_{FWE}<0.05$

Supplementary Table S4. Regions activated in migraine/control groups during reward consumption

Contrast	Groups	Cluster size (voxel)	Peak T-value	Coordinates (MNI) x	y	z	Region	Hemisphere
Gain-neutral outcome	Migraine	3819	8.60	-36	-79	-16	Fusiform gyrus	Left
			7.79	-24	-82	-13		
			7.71	-42	-67	-19		
			8.13	12	-82	-13	Lingual gyrus	Right
			7.80	36	-79	-13	Inferior occipital gyrus	Right
			7.74	30	-85	11	Middle occipital gyrus	Right
			7.51	30	-88	17		
			7.45	-9	-94	-7	Calcarine sulcus	Left
			6.94	-9	-97	-1		
			7.24	30	-61	-13	Fusiform gyrus	Right
			6.98	33	-58	-16		
			6.88	36	-67	-16		
			7.09	-15	-85	-76	Lingual gyrus	Left
			6.12	0	-73	2		
			6.82	15	-94	11	Cuneus	Right
			6.57	-21	-97	5	Middle occipital gyrus	Left
		607	6.69	3	47	11	Anterior Cingulum	Right
			5.91	18	38	53	Superior frontal gyrus	Right
			4.19	15	32	59		
			4.09	18	53	38		
			5.65	-21	50	41		Left
			5.14	-15	53	41		
			4.56	-12	56	38		
			5.25	0	62	14	Medial superior frontal gyrus	Left
			4.85	-9	44	53		
			4.75	0	62	26		
			4.61	-6	56	38		
			4.56	-9	53	41		
			4.51	-3	59	35		
			4.68	3	59	32		Right
			4.07	15	26	62		
		137	6.12	48	17	-31	Middle temporal pole	Left
			5.71	45	14	-34		Right
			5.20	39	23	-19		Right
			5.19	42	23	-13		

			4.82	48	35	-13	Orbital inferior frontal gyrus	
			3.83	51	38	-10		
		113	5.65	-30	14	-19	Insula	Left
			5.64	-51	11	-31	Middle temporal pole	Left
			4.92	-54	8	-28	Middle temporal gyrus	Left
			4.55	-54	2	-31		
			4.16	-30	11	-31	Superior temporal pole	Left
			3.53	-42	20	-13	Orbital inferior temporal pole	Left
	Control	9198	16.41	-15	-88	-10	Lingual gyrus	Left
			12.22	-27	-82	-16		
			9.62	-3	-67	-1		
			14.37	15	-91	-4		Right
			14.18	15	-88	-10		
			13.71	18	-85	-13		
			10.85	6	-76	2		
			8.50	6	-43	2		
			15.68	21	-91	5	Superior occipital gyrus	Right
			10.75	-24	-88	23		Left
			15.48	-12	-94	-1	Middle occipital gyrus	Left
			13.40	-18	-94	11		
			10.65	-39	-85	2		
			13.75	27	-67	-13	Fusiform gyrus	Right
			10.48	6	-82	2	Calcarine	Right
			8.58	0	59	8	Medial superior frontal gyrus	Left
Success-neutral outcome	Migraine	2344	7.29	15	-82	-13	Lingual gyrus	Right
			5.32	12	-52	2		
			5.48	12	-52	2		
			5.19	9	-73	-4		
			6.15	-18	-85	-16		Left
			5.82	-3	-73	2		
			6.59	-24	-79	-13	Fusiform gyrus	Left
			6.49	-36	-79	-16		
			5.52	-42	-67	-19		
			6.42	-9	-94	-7	Calcarine sulcus	Left
			6.26	0	-67	14		
			5.51	-12	-58	5		
			5.32	12	-85	2		Right
			5.68	3	-76	17	Cuneus	Right
			5.23	18	-91	11		

		5.19	21	-94	17	Superior occipital gyrus	Right
		5.17	-9	-94	17		Left
	82	5.72	-27	11	-19	Insula	Left
		5.65	-30	14	-16		
		4.92	-48	14	-31	Middle temporal pole	Left
		4.41	-48	8	-34		
	181	5.31	0	53	11	Medial superior frontal gyrus	Left
	72	5.13	48	17	-31	Middle temporal pole	Right
		4.67	45	17	-16	Superior temporal pole	Right
Control	5394	15.39	-12	-91	-10	Lingual gyrus	Left
		12.14	15	-91	-4		
		8.23	-12	-46	-1		
		12.66	15	-91	-4		Right
		8.51	9	-43	2		
		13.44	-15	-94	-1	Middle occipital gyrus	Left
		10.84	-21	-94	11		
		7.49	-39	-85	-1		
		11.32	21	-91	5	Superior occipital gyrus	Right
		7.45	-24	-88	23		Left
		8.90	-33	-70	-16	Fusiform gyrus	Left
		8.24	24	-73	-13		Right
		8.29	21	-28	-10	Hippocampus	Right
		8.04	33	23	-16	Orbital inferior frontal gyrus	Right
		7.45	-39	-82	-7	Inferior occipital gyrus	Left
	958	8.04	-3	62	8	Medial superior frontal gyrus	Left
		6.90	-3	62	17		
		5.06	-9	47	47		
		3.56	0	41	38		
		5.17	9	41	53		Right
		4.46	6	53	44		
		3.81	6	59	32		
		6.28	3	47	14	Anterior cingulum	Right
		6.05	18	35	50	Superior frontal gyrus	Right
		5.38	15	29	53		
		5.00	-15	44	44		Left
		4.22	-12	53	38		
		3.92	-12	35	53		

Note. Cluster-level $p_{FWE}<0.05$

Supplementary Table S5. Regions activated in migraine/control groups during loss consumption

Contrast	Groups	Cluster size (voxel)	Peak T-value	Coordinates (MNI) x	y	z	Region	Hemisphere
Loss-neutral outcome	Migraine	536	5.59	15	-82	-13	Lingual gyrus	Right
			5.42	-6	-91	-7	Calcarine sulcus	Left
			4.90	12	-97	2		Right
			5.22	-27	-76	-16	Fusiform gyrus	Left
			5.07	21	-76	-16		Right
			4.87	18	-94	14	Cuneus	Right
	Control	2332	12.85	-15	-88	-10	Lingual gyrus	Left
			5.64	-18	-55	2		
			10.49	15	-82	-10		Right
			5.95	9	-43	-1		
			12.64	-12	-94	-1	Middle occipital gyrus	Left
			9.15	-18	-94	11		
			7.26	30	-88	14		Right
			8.81	15	-94	11	Cuneus	Right
			7.03	15	-91	23		
			5.90	12	-88	29		
			5.86	6	-88	26		
			5.75	-9	-91	26		Left
			5.08	-6	-88	29		
			5.72	-15	-58	5	Calcarine sulcus	Left
			5.55	-12	-61	8		
			5.21	18	-34	-10	Parahippocampal gyrus	Right
		267	7.01	39	23	-16	Orbital inferior frontal gyrus	Right
			6.94	33	20	-13	Insula	Right
			4.86	48	17	-31	Middle temporal pole	Right
			4.83	51	17	-25	Superior temporal pole	Right
			4.77	54	26	11	Inferior frontal gyrus triangularis	Right
			4.23	60	20	14	Inferior frontal gyrus opercularis	Right
Failure-neutral outcome	Migraine	154	5.23	18	-82	-13	Lingual gyrus	Right
			3.53	12	-67	-7		
		169	4.81	-24	-79	-16	Lingual gyrus	Left
			4.48	-15	-79	-13		

		4.56	-9	-91	-7	Calcarine sulcus	Left
	3288	12.20	-15	-88	-4	Lingual gyrus	Left
		7.05	-9	-46	-1		
		6.84	-18	-55	2		
		11.06	12	-79	-10		Right
		9.85	12	-91	-4		
		7.72	9	-43	-1		
		11.33	-12	-94	-1	Middle occipital gyrus	Left
		8.42	-18	-91	14		
		8.34	-21	-94	11		
		9.67	-24	-79	-13	Fusiform gyrus	Left
		9.25	3	-88	-7	Calcarine sulcus	Left
		6.63	-15	-58	5		
		8.94	21	-91	5	Superior occipital gyrus	Right
		8.55	24	-91	14		
		8.04	21	-91	20		
		8.70	18	-94	14	Cuneus	Right
	133	6.94	-54	2	-19	Middle temporal gyrus	Left
		3.97	-51	-22	-10		
		4.42	-42	2	-19	Superior temporal pole	Left
Control	471	6.50	33	20	-13	Insula	Right
		5.99	42	20	-19	Orbital inferior frontal gyrus	Right
		5.14	42	29	-13		
		5.70	51	17	-25	Superior temporal pole	Right
		5.65	48	14	-31	Middle temporal pole	Right
		5.28	60	20	11	Inferior frontal gyrus opercularis	Right
		5.23	57	2	-13	Superior temporal gyrus	Right
		4.40	54	-7	-13		
		5.14	42	2	-37	Inferior temporal gyrus	Right
		3.98	57	23	2	Inferior frontal gyrus triangularis	Right
		3.73	30	8	-25	Superior temporal pole	Right
	100	4.31	3	53	14	Medial superior frontal gyrus	Right
		4.16	3	53	5		
		3.70	3	59	2		

Note. Cluster-level p_{FWE}<0.05

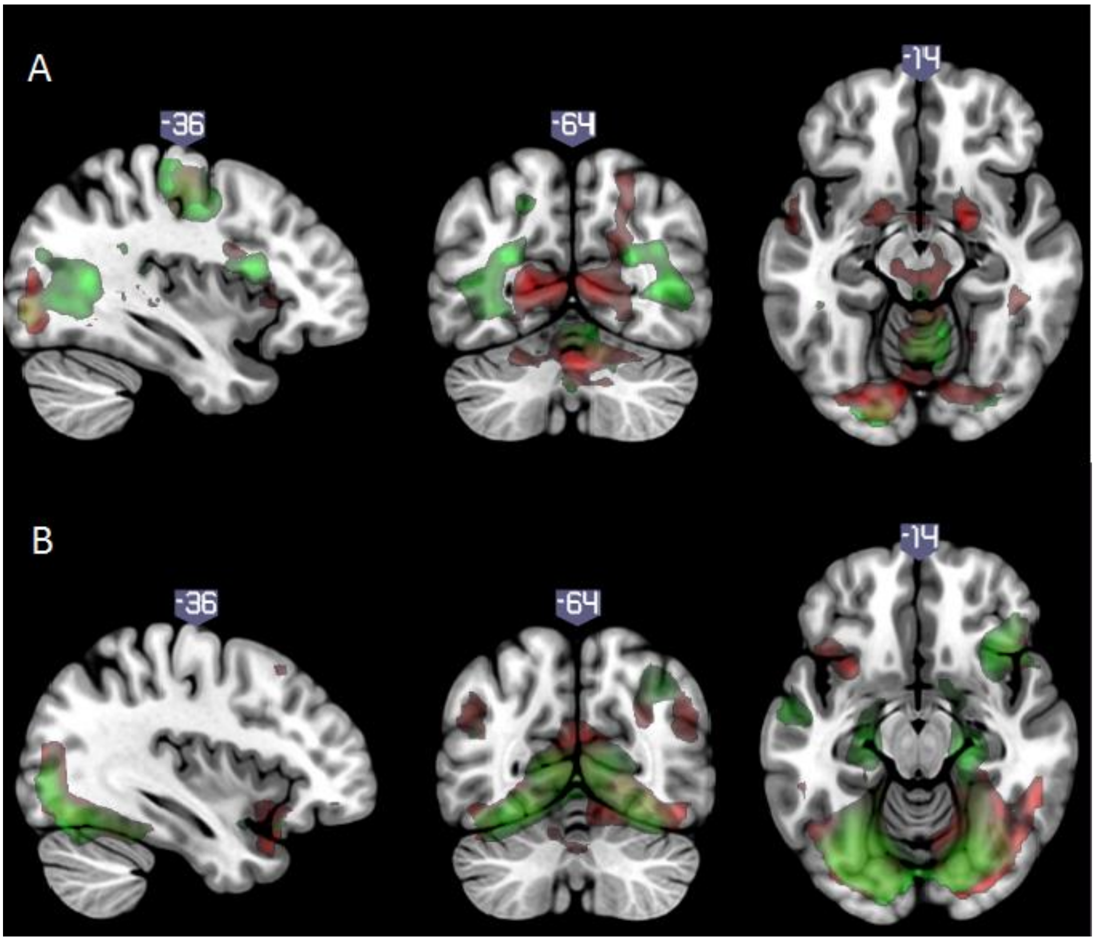

Supplementary Figure S7. Overview of activated regions during reward processing in migraineurs and healthy controls. A: Activations yielded for reward anticipation (win-neutral cue) contrast are presented: Red= migraine group, green=control group. B: Activations yielded for reward consumption (gain-neutral outcome/success-neutral outcome) contrasts: Red= migraine group, green=control group. The displayed brain regions are based on the findings presented in Supplementary Table S2-S4.

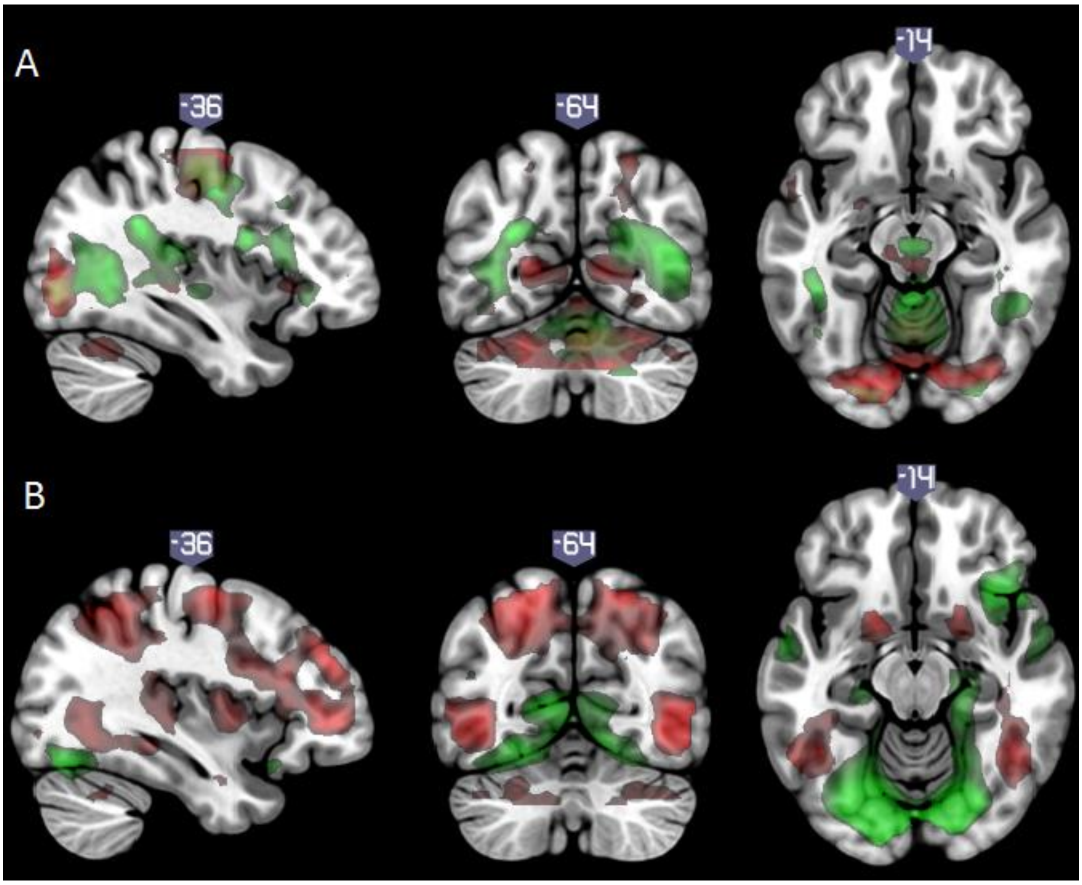

Supplementary Figure S8. Overview of activated regions during loss processing in migraineurs and healthy controls. A: Activations yielded for loss anticipation (loss-neutral cue) contrast are presented: Red= migraine group, green=control group. B: Activations yielded for loss consumption (loss-neutral outcome/failure-neutral outcome) contrasts: Red= migraine group, green=control group. The displayed brain regions are based on the findings presented in Supplementary Table S1-S3.

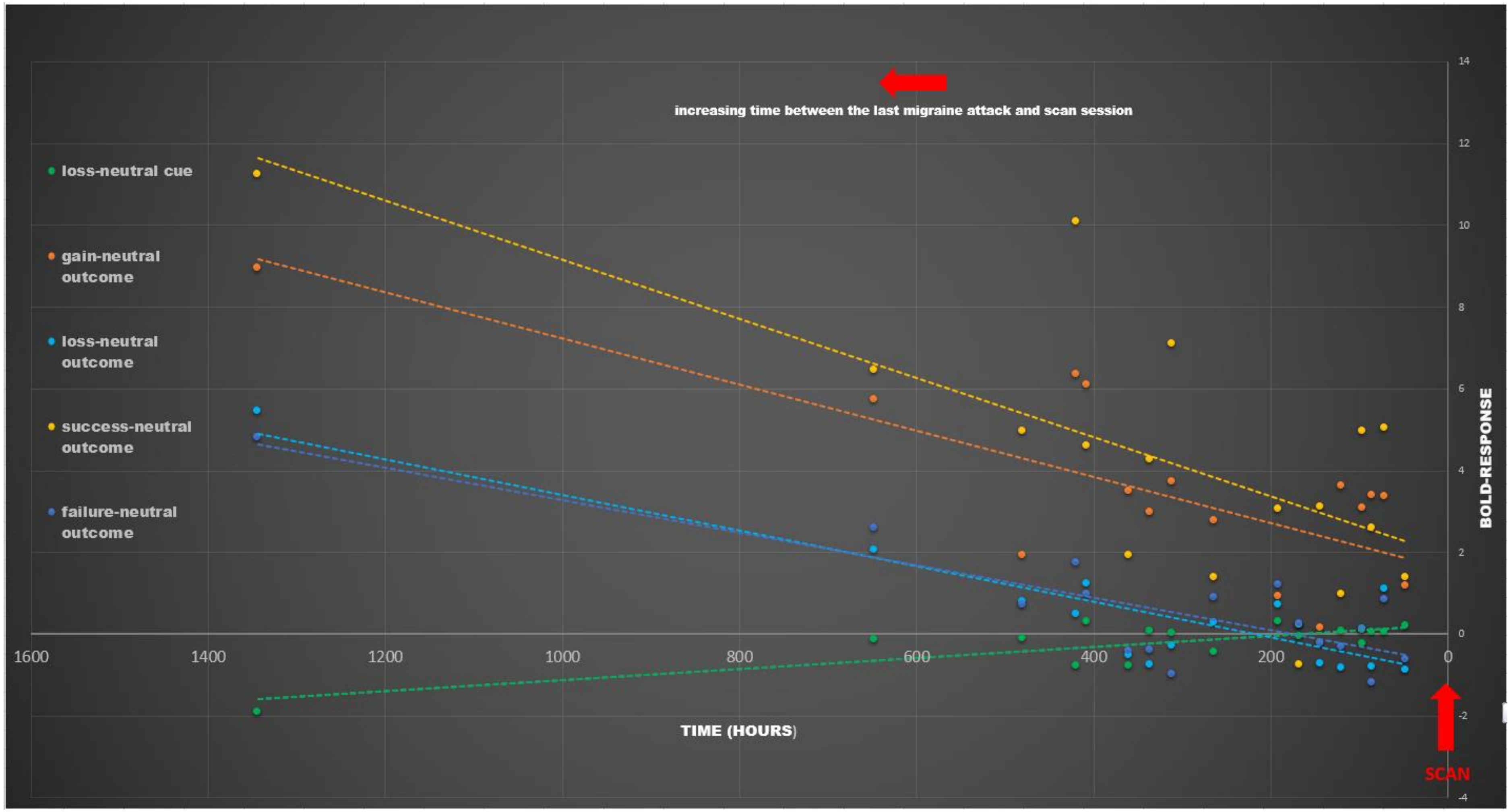

Supplementary Figure S9. BOLD-responses yielded for loss anticipation and reward/loss consumption in relation to the time of the last migraine attack for illustrative purposes. Green: activation of right hippocampus (MNI: x=15 y= -10 z= -19) to loss-neutral cue; Orange: activation of right fusiform gyrus (MNI: x=36 y= -76 z= -16) to gain-neutral outcome; Yellow: activation of right fusiform gyrus (MNI: x=24 y=-76 z=-13) to success-neutral outcome; Light blue: activation of left calcarine (MNI: x= -27 y= -61 z=5) to loss-neutral outcome; Dark blue: activation of left calcarine (MNI: x= -18 y= -52 z=5) to failure-neutral outcome.
